# Effects of different levels of physical activity on the health-related quality of life among rural junior high school students in China: the moderating role of parental co-participation in physical activities

**DOI:** 10.3389/fpubh.2025.1556246

**Published:** 2025-05-23

**Authors:** Weilin Yang, Zhiyun Zhao, Pengcheng Gao, Xiaodan Guo, Xuguang Jia, Marcin Białas

**Affiliations:** ^1^Doctor School, Gdansk University of Physical Education and Sport, Gdansk, Poland; ^2^School of Sports and Health, Yibin University, Yibin, China; ^3^School of Education, University of Malaya, Kuala Lumpur, Malaysia; ^4^Postgraduate School, Pukyong National University, Pusan, Republic of Korea; ^5^The Third People's Hospital of Yibin, Yibing, China

**Keywords:** physical activity, health-related quality of life, parental co-participation, rural junior high school students, China

## Abstract

**Background:**

Health benefits are associated with physical activity (PA) and PA levels. This study aims to explore the impact of PA levels (low, moderate, high) on health-related quality of life (HRQoL) among rural junior high school students in China, as well as the moderating role of parental co-participation in physical activities.

**Methods:**

A stratified cluster sampling method was used to select 1,440 junior high school students from rural areas in southwest China. A total of 1,181 students completed the questionnaire, with a completion rate of 82.0%, including 608 boys (51.45%). HRQoL was measured using the Chinese version of the PedsQL 4.0, and PA levels was analyzed using the IPAQ-SF. *T*-tests, chi-square tests, linear regression, and two-way ANOVA were used for statistical analysis.

**Results:**

The results showed that 81.8% of rural junior high school students participated in moderate PA and high PA, with 82.3% of boys and 81.2% of girls. In both unadjusted and adjusted models, compared to low PA, students participating in moderate PA and high PA had significantly higher HRQoL scores in all dimensions, summary scales, and total scales, with the high PA showing particularly higher scores. Parental co-participation significantly moderated the relationship between PA and HRQoL (*F* = 13.569, *p* < 0.001), and the moderating effect was significant at every PA level, especially at high PA here the effect was most prominent.

**Conclusion:**

Physical activity has a significant positive impact on HRQoL of rural junior high school students, with higher PA levels leading to more significant improvements in all HRQoL dimensions, summary scales, and total scales. Parental co-participation enhances the positive effect of PA on the HRQoL of rural junior high school students, particularly at the high PA, where the moderating effect is more pronounced. Parental co-participation, through the provision of social support, emotional reinforcement, and behavioral modeling, significantly enhances adolescents’ intrinsic motivation to participate in physical activity, and further augments its salutary effects on their physical and psychological well-being. This study provides empirical support for health promotion among rural adolescents.

## 1 Introduction

Physical activity (PA) has a broad and profound impact on the health of adolescents, not only improving physical health but also enhancing psychological well-being and overall health-related quality of life (HRQoL) (1–6). HRQoL is a subjective and multidimensional concept that encompasses various aspects such as physical, psychological, social, and school functions (7, 8). In recent years, HRQoL in adolescents has become an important health outcome, as it can measure the risk of disease precursors and indicate the health status of the next generation (9). Particularly for adolescents undergoing growth and development, PA is considered an important means to improve HRQoL (10). However, youth activity levels have remained low in countries around the world (11, 12), posing a global public health challenge (13). China was one of the countries where these related problems were increasingly serious. For instance, studies indicated that only 34.1% of adolescents in China comply with moderate-to-vigorous physical activity guidelines, and this figure has continued to decrease, putting the health of China’s youth at risk (14). Some studies suggested that physical inactivity was associated with obesity and overweight, impaired cognitive function, and mental health (15, 16), further affecting the HRQoL of adolescents. However, the relationship between PA levels and HRQoL may be influenced by various family factors (17, 18), among which parental co-participation, as one of the key family factors, may play a moderating role. Although some studies have indicated that high PA can improve mental health in adolescents, recent evidence suggests that its effect on anxiety reduction is limited (19, 20). Research suggests that parental co-participation can enhance the positive effects of PA on health by providing social support, emotional, encouragement, and role modeling (21–23).

In rural areas, adolescent health issues are increasingly drawing attention, especially as rural adolescents generally engage in lower levels of PA due to the lack of sports facilities and opportunities to participate in PA. Compared to urban adolescents, rural adolescents rely more on outdoor activities and daily labor for PA (24, 25). Moreover, due to limited economic conditions and educational resources, rural parents vary in their emphasis on their children’s PA (25). Rural family structures are relatively traditional, with parents playing a significant role in their children’s lives. Parental co-participation not only provides emotional support to adolescents but also helps them establish good habits, enhance self-confidence, and develop social skills by directly involving in their PA (26–28). However, although the role of parental co-participation in PA has been mentioned in some studies, research on how parental co-participation moderates the impact of PA on HRQoL remains limited, especially in rural areas.

The level of PA also affects HRQoL outcomes in different ways. Low PA typically helps with relaxation and maintaining health, but its effect on improving HRQoL in adolescents is relatively limited. Moderate PA and high PA, on the other hand, are considered to significantly improve adolescents’ physical health and psychological well-being (29–31). However, the effects of these PA may differ when parental co-participation is involved. Parental co-participation may help children better cope with the challenges of PA by providing emotional and psychological support, thereby further enhancing their HRQoL.

**Figure 1:**
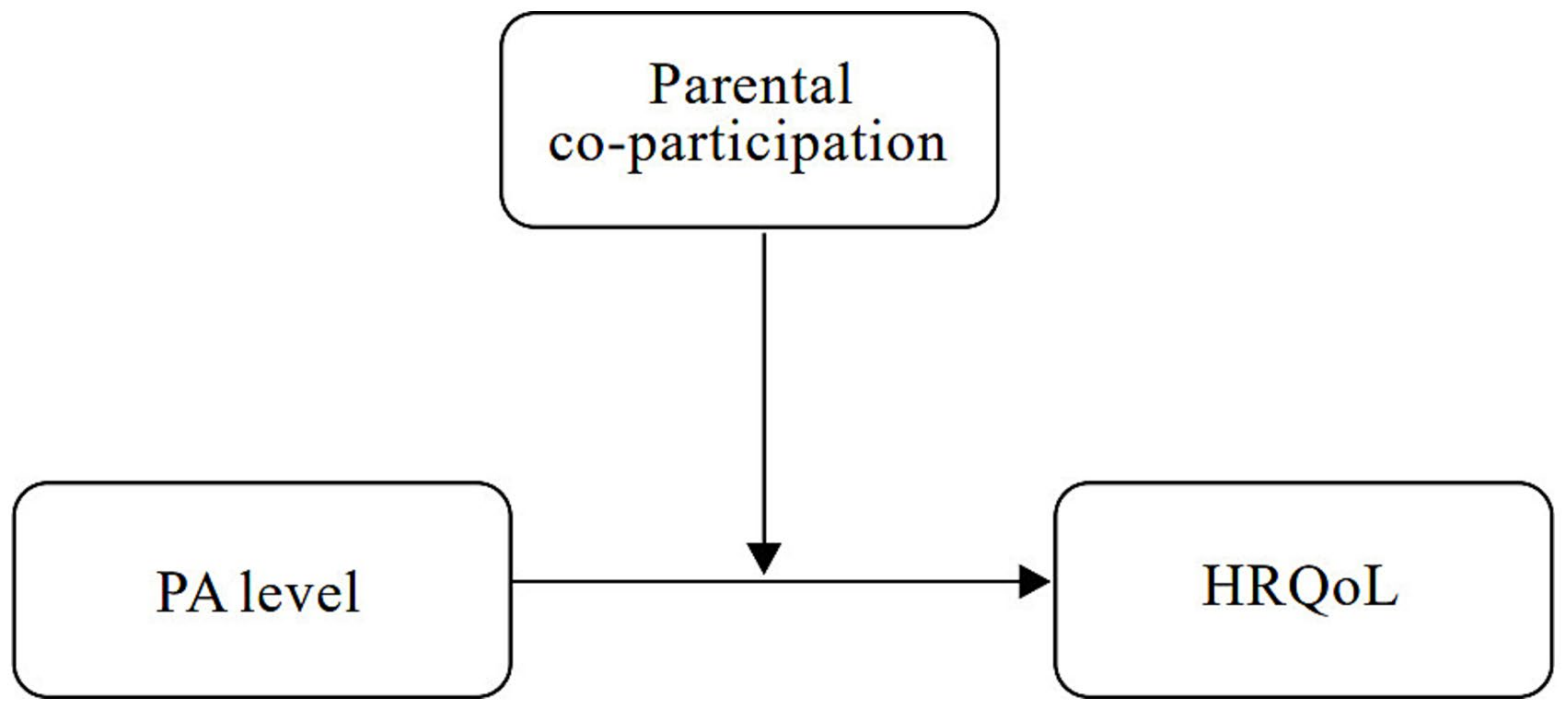
The moderating effect path hypothesis.

Based on the above background, this study aims to investigate the effect of PAon the HRQoL among junior high school students in rural China, with a particular focus on the moderating role of parental co-participation in physical activities in the PA–HRQoL relationship (Figure 1). We hypothesize that parental co-participation moderates the association between PA and HRQoL among rural adolescents. Higher PA levels, together with parental support, are associated with greater HRQoL improvements.

By addressing these questions, this study hopes to provide theoretical support and practical guidance for health promotion strategies for rural adolescents in China, particularly emphasizing the key role of parental co-participation in enhancing the effects of PA on HRQoL.

## 2 Materials and methods

### 2.1 Participants

This cross-sectional study was conducted in rural junior high schools in southwest China from March to April 2024, using a stratified cluster sampling method to enhance the representativeness of the sample. First, the southwestern region was stratified based on regional economic development levels (with per capita gross domestic product as the primary reference indicator), disparities in urban–rural educational resources, and geographic distribution. First, eight counties were selected: Jiang’an County and Gao County in Sichuan Province, Xishui County and Dafang County in Guizhou Province, Huize County and Luoping County in Yunnan Province, and Wushan County and Yunyang County in Chongqing Municipality. Then, eight township junior high schools were randomly selected from these counties. Finally, two classes from grades 7, 8, and 9 were randomly selected from each school (approximately 30 students per class). Each school had six classes surveyed, resulting in a total of 1,440 junior high school students participating in the study.

The researchers received training before conducting the survey. On the day of the survey, students completed the questionnaire independently in the classroom, and the researchers provided explanations if students encountered any semantic or conceptual difficulties during the completion of the questionnaire.

In total, 1,239 junior high school students submitted completed questionnaires, with a response rate of 80.0%. Thirty questionnaires were excluded due to incomplete information on PA, 10 were excluded due to missing data on parental co-participation in physical activities, and 18 were excluded due to missing PedsQL data. Finally, 1,181 junior high school students were included in the analysis, of whom 608 were boys and 573 were girls.

Prior to the study, the research objectives were explained to schools, parents or guardians, and students. Written informed consent was obtained from all participants or from their legal representatives to ensure voluntary participation. This study was approved by the Ethics Committee of Psychology at the Third Hospital of Yibin City, Sichuan, China (Approval No.: YBSDSRMYY-2023-02).

### 2.2 Measures of physical activity levels

The PA was assessed using the International Physical Activity Questionnaire-Short Form (IPAQ-SF) (32). The Chinese-version has been widely used in children, adolescents, and adults in China, the reliability and validity of the scale have been previously verified in the relevant population (Cronbach’s *α* = 0.79) (33, 34) (Supplementary File 1). Based on different types of PA, the IPAQ categorizes PA into three levels: walking (shown here as light activity), moderate activity and vigorous activity. To quantify the energy expenditure of these activities, IPAQ assigns a corresponding metabolic equivalent of task (MET) coefficient. Specifically, the MET coefficients are 3.3 for light activity, 4.0 for moderate activity, and 8.0 for vigorous activity. These coefficients serve as indicators of the relative energy expenditure associated with each level of PA, and the total PA is calculated using the following formula:

Total MET min/week = Light (3.3*min*days) + Moderate (4.0*min*days) + Vigorous (8.0*min*days).

According to the IPAQ criteria, PA is classified as “high PA” if a person engages in a combination of light, moderate, or vigorous activity for 7 or more consecutive days, with a total PA of ≥3,000 MET-min/week. “Moderate PA” requires light, moderate, or vigorous activity for 5 or more days, with a total PA of ≥600 MET-min/week. Total PA of less than 600 MET-min/week is classified as “low PA.” Based on these criteria, participants were categorized into a high PA (HPA) group, a moderate PA (MPA) group, and a low PA (LPA) group (34).

### 2.3 Measures of health-related quality of life

The Pediatric Quality of Life Inventory 4.0 (PedsQL 4.0) was used to assess the HRQoL of adolescents. This multidimensional tool consists of 23 items that cover four domains: physical functioning (8 items), emotional functioning (5 items), social functioning (5 items), and school functioning (5 items) (35–38). The Chinese Version internal consistency reliability for total scale score (Cronbach’s *a* = 0.90), Physical health summary score (Cronbach’s *a* = 0.81), and psychosocial health summary score (Cronbach’s a = 0.89) were excellen (39) (Supplementary File 2). Each item is rated on a five-point Likert scale ranging from 0 to 4 (0 = never a problem, 1 = almost never a problem, 2 = sometimes a problem, 3 = often a problem, and 4 = almost always a problem). To calculate HRQoL scores, items are reverse-scored and transformed linearly to a 0–100 scale (0 = 100, 1 = 75, 2 = 50, 3 = 25, 4 = 0), with higher scores indicating better HRQoL. The scale score is calculated by dividing the sum of the item scores by the number of items answered (this accounts for missing data). If more than 50% of the items in a scale are missing, the scale score is not calculated. The score for each domain is calculated by adding the item scores within that domain and dividing by the number of items in that domain (40). Additionally, the PedsQL 4.0 provides a psychosocial functioning summary scale (calculated as the mean of the emotional, social, and school subscale items) and a total functioning summary scale (calculated as the mean of all items) (40).

### 2.4 Parental co-participation

Parental co-participation in physical activities was assessed through a structured self-administered questionnaire. The specific question asked was: “Do your parents accompany you in your daily life and leisure PA?” The response options were: (1) both parents accompany, (2) only father accompanies, (3) only mother accompanies, and (4) others accompany. For the purpose of this study, participants were categorized as having parental co-participation (both parents, only father, or only mother) or not having parental co-participation (others accompany) based on the research requirements (41).

### 2.5 Control variables

This study also considered a series of individual and parental factors that could influence PA levels and HRQoL based on previous research (42). These variables included two main aspects; socio-demographic characteristics and parental factors. First, sociodemographic characteristics included age, gender, grade, ethnicity, and siblings. Second, parental factors primarily focused on the education levels of the father and mother. Since rural parents generally have lower education levels, this study classified parental education as “primary school or below” and “junior high school or above.” Detailed settings for each variable are provided in Table 1.

**Table 1:** Coding of variables.

Variable	Coding
PedsQL 4.0	Never a problem = 0, Almost never a problem = 1, Sometimes a problem = 2, Often a problem = 3, Almost always a problem = 4
Parental co-participation	Parents = 1, Father = 2, Mother = 3, Other = 4
Age	12 ~ 15 years old
Gender	Boy = 1, girl = 2
Grade	7th Grade = 1, 8th Grade = 2, 9th Grade = 3
Ethical group	Han = 1, Non-Han = 2
Siblings	Yes = 1, No = 2
Father’s education	Primary school or below = 1, Junior high school or above = 2
Mother’s education	Primary school or below = 1, Junior high school or above = 2

### 2.6 Statistical analysis

Statistical data analysis was performed using the SPSS software package (version 29, Armonk, NY: IBM Corp). Skewness and kurtosis were used to determine the normality of the variables of interest. The skewness and kurtosis values for HRQoL and its sub-dimensions ranged from −1 to 1, indicating the suitability for testing and regression analysis (8). Descriptive statistics were presented using numbers and percentages for categorical variables and means with standard deviations for continuous variables. *T*-tests or chi-square tests were used to analyze the differences in characteristics between boys and girls. Regression analysis was conducted to examine the relationship between different levels of PA and HRQoL among adolescents, while two-way ANOVA was used to test the moderating role of parental co-participation on the impact of PA on HRQoL. In view of the increased risk of type I errors associated with multiple hypothesis testing, the Benjamini-Hochberg procedure was employed to adjust the original *p*-values, thereby controlling the overall false discovery rate and ensuring the robustness of statistical inference. Statistical significance for all tests was set at *p* < 0.05.

## 3 Results

### 3.1 Descriptive statistics

**Figure 2:**
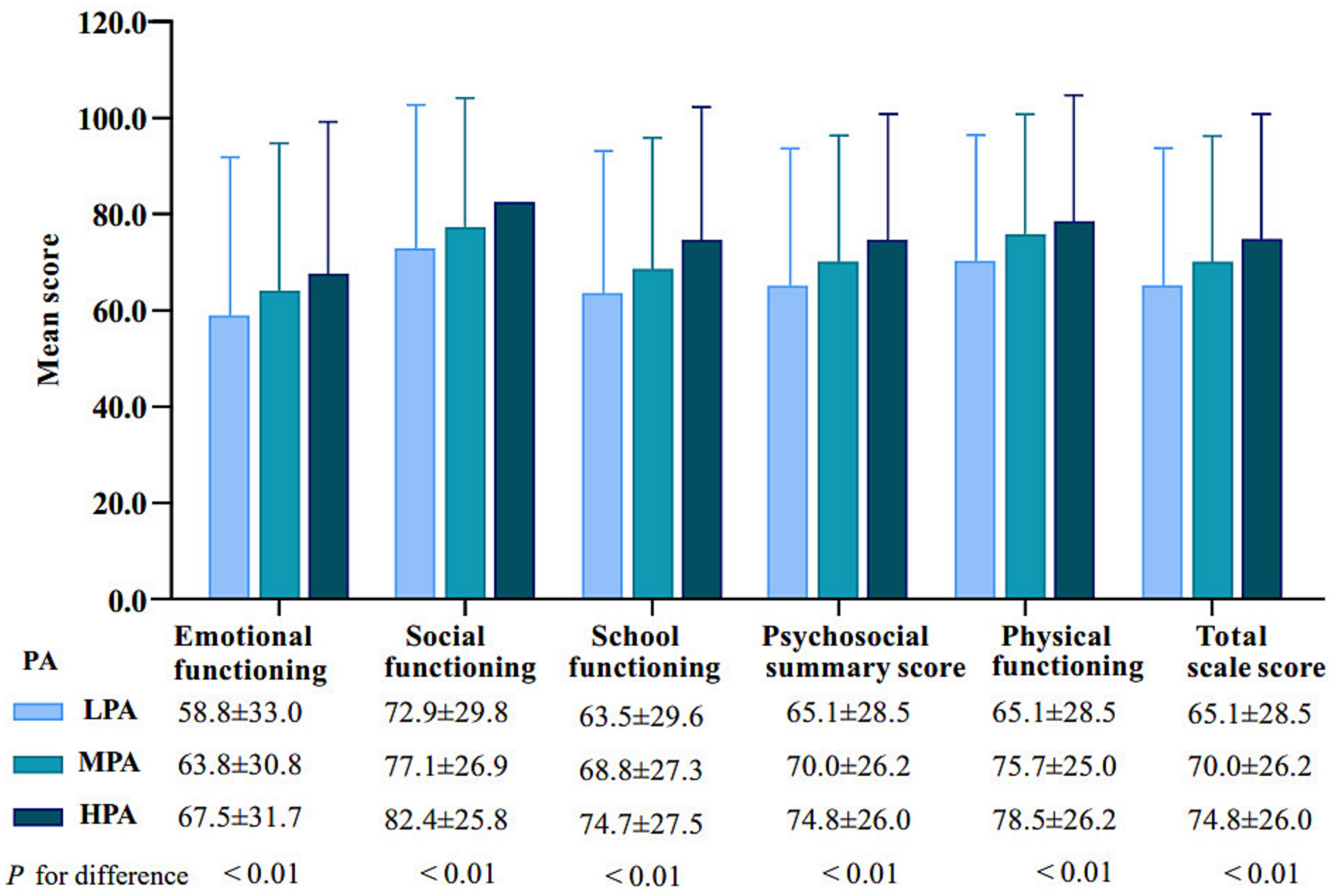
According to PA levels and HRQoL score.

A total of 1,181 rural middle school students were recruited for statistical analysis in this study, including 608 boys (51.45%) and 573 girls (48.51%). The descriptive statistics based on gender (Table 2). The descriptive analysis shows that the average age of the middle school students was 13.5 years, with 7th grade students accounting for 48.7%, 8th grade students for 26.8%, and 9th grade students for 24.3%. Most of the students were Han ethnicity (82.6%), and 47.0% had siblings. The education levels of fathers and mothers were middle school or above for 91.9 and 43.7%, respectively. Regarding PA, 81.8% of the students participated in MPA and HPA with 82.3% of boys and 81.2% of girls meeting this level. Additionally, the average total HRQoL score was 83.1, with average scores for Emotional Functioning, Social Functioning, School Functioning, Psychosocial Summary Score, and Physical Functioning being 77.4, 87.7, 79.6, 87.3 and 81.6, respectively. *T*-tests or chi-square tests showed significant gender differences in PA, HRQoL, and father’s education level (*p* < 0.05). Moreover, as PA levels increased, HRQoL scores across all dimensions, summary scales, and total scale significantly improved (Figure 2).

**Table 2:** Demographic characteristics of participants, *n* = 1,181.

Variables	Total	Boys	Girls	*p*-value
Age (Year)^a^	13.5 (0.7)	13.5 (2.6)	13.4 (2.6)	0.05
Grade (*n*)^b^				0.08
7th Grade	576 (48.7)	282 (46.3)	294 (51.3)	
8th Grade	317 (26.8)	168 (27.6)	149 (26.0)	
9th Grade	288 (24.3)	158 (25.9)	130 (22.6)	
Ethical group (*n*)^b^				0.14
Han	976 (82.6)	512 (84.2)	464 (80.9)	
Non-Han	205 (17.3)	96 (15.7)	109 (19.0)	
Siblings (*n*)^b^				0.45
Yes	556 (47.0)	280 (46.0)	276 (48.1)	
No	625 (52.9)	328 (53.9)	297 (51.8)	
Father’s education (*n*)^b^				0.01
Primary school or below	95 (8.0)	60 (9.8)	35 (6.1)	
Junior high school or above	1,086 (91.9)	548 (90.1)	538 (93.8)	
Mother’s education (*n*)^b^				0.30
Primary school or below	664 (56.2)	333 (54.7)	331 (57.7)	
Junior high school or above	517 (43.7)	275 (45.2)	242 (42.2)	
PA (*n*)^b^				
LPA	214 (18.1)	107 (17.6)	107 (18.6)	0.00
MPA	349 (29.5)	176 (28.9)	173 (30.1)	
HPA	618 (52.3)	325 (53.4)	293 (51.1)	
PedsQL^a^				
Emotional functioning	77.4 (21.3)	80.4 (19.9)	74.2 (22.3)	0.00
Social functioning	87.7 (15.7)	89.2 (15.2)	86.1 (16.1)	0.00
School functioning	79.6 (19.1)	81.3 (18.8)	77.7 (19.3)	0.00
Psychosocial summary score	87.3 (14.5)	83.6 (15.5)	79.4 (16.6)	0.00
Physical functioning	81.6 (16.2)	89.3 (13.35)	85.3 (15.5)	0.00
Total scale score	83.1 (14.8)	85.1 (14.0)	80.8 (15.3)	0.00

PA, physical activity; LPA, low physical activity; MPA, moderate physical activity; HPA, high physical activity.

^a^Data are presented as mean (SD).

^b^Data are presented as number (%).

### 3.2 Regression model results

The relationship between different PA levels and various dimensions of HRQoL (Table 3). In both crude and adjusted analyses, LPA was used as the reference group. The results demonstrated that higher PA levels were associated with higher HRQoL scores across all dimensions. Particularly, HPA significantly outperformed LPA across all dimensions. MPA also had a significant positive impact across all dimensions compared to LPA, although the effect was smaller than HPA. The results of the adjusted analyses, which included personal and parental characteristics, were generally consistent with those of the crude analyses, indicating that the impact of different PA levels on HRQoL in rural middle school students was robust.

**Table 3:** Regression analysis of PA levels and HRQoL.

HRQoL	PA	Crude analysis	Adjusted analysis ^a^
		β^b^(95% CI)	β^b^ (95% CI)
Emotional functioning	LPA	Reference group	
	MPA	4.99 (0.70; 9.27)*	4.90 (0.73; 9.21)*
	HPA	8.63 (3.79; 11.47)**	8.30 (3.65;13.33)**
Social functioning	LPA	Reference group	
	MPA	4.37 (0.65; 8.09)*	4.35 (0.65; 8.10)*
	HPA	9.47 (5.26; 12.67)**	9.52 (5.25; 13.75)**
School functioning	LPA	Reference group	
	MPA	5.27 (1.48; 9.07)**	5.49 (1.72; 9.27)**
	HPA	11.32 (6.84; 14.41)**	11.56 (7.22; 15.83)**
Psychosocial summary score	LPA	Reference group	
	MPA	4.88(1.25; 8.51)**	4.94 (1.34; 8.55)**
	HPA	9.74(5.64; 12.84)**	9.80 (5.72; 13.95)**
Physical functioning	LPA	Reference group	
	MPA	5.45 (1.98; 8.92)**	5.59 (2.13; 9.05)**
	HPA	8.21 (4.29; 12.13)**	8.44 (4.50; 12.38)**
Total scale score	LPA	Reference group	
	MPA	5.02 (1.55; 8.49)**	5.11 (1.65; 8.56)**
	HPA	9.36 (5.47; 13.2)**	9.49 (5.55; 13.42)**

HRQoL, Health-related quality of life; LPA, low physical activity; MPA, moderate physical activity; HPA, high physical activity.

^a^Adjusted models were controlled for the characteristics of junior high school students (Gender, grade, sibling status, ethnicity) and parental characteristics (level of education).

^b^Unstandardized β coefficients with 95% CIs *P < 0.05, **p < 0.01.

### 3.3 Moderation effect analysis

**Figure 3:**
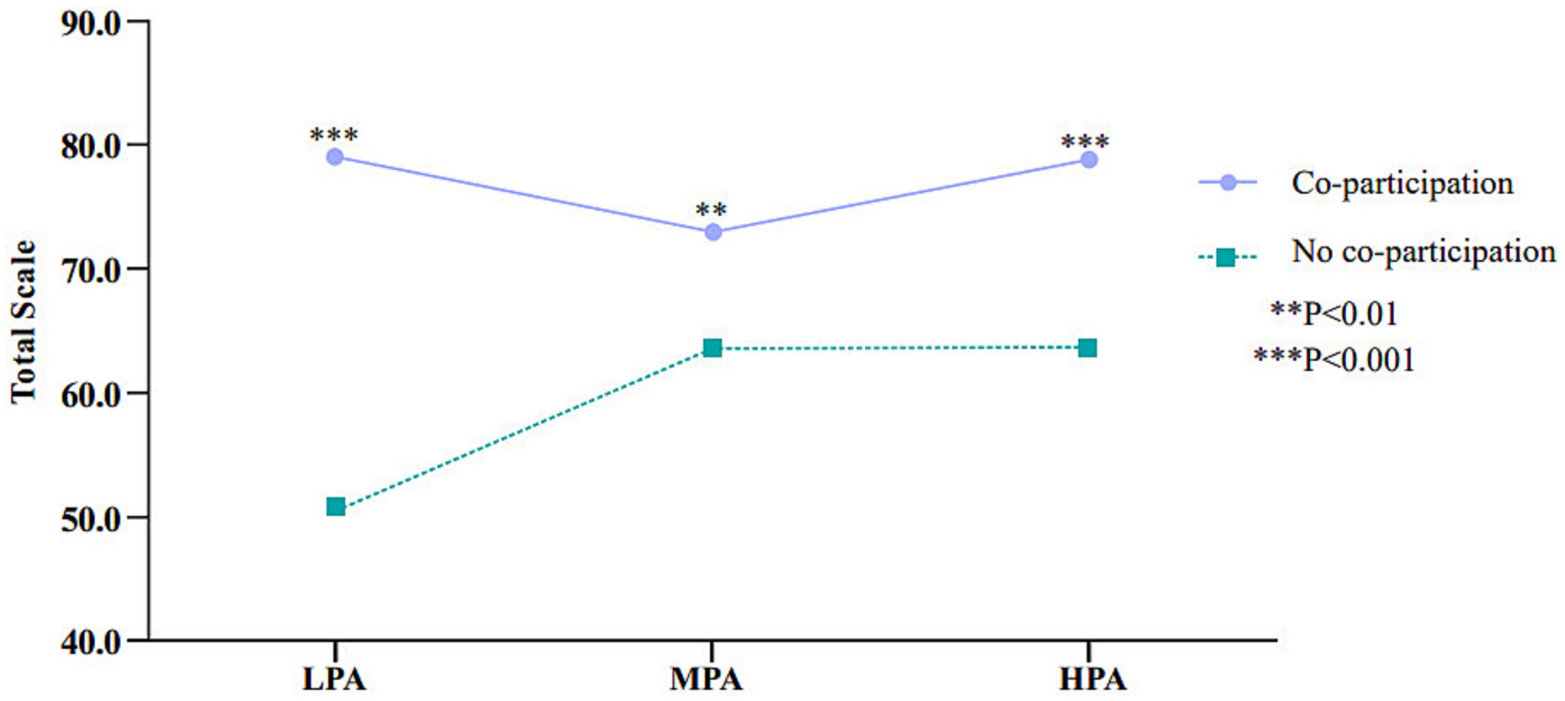
The moderating effect of parental co-participation on PA levels and HRQoL.

The moderating effect of parental co-participation on the relationship between PA and total HRQoL (Table 4 and Figure 3). The interaction between PA and parental co-participation had a significant impact on total HRQoL (*F* = 13.569, *p* < 0.001), indicating that parental co-participation is a significant moderator in the relationship between PA and total HRQoL. At different PA levels, the group without parental co-participation showed an increase in total HRQoL scores as PA levels increased, but the growth was relatively gradual. In contrast, the group with parental co-participation had higher scores at all PA levels, and the higher the PA level, the stronger the positive impact of parental co-participation on HRQoL, especially at the HPA, where the moderating effect was particularly pronounced.

**Table 4:** Moderating effect.

Model	No co-participation	Co-participation	SE	*t*	*P*
Model 1(*R*^2^ = 0.130)					
LPA	50.7(25.2)	79.0(18.1)	2.582	−10.978	*P* < 0.001
MPA	63.3(24.5)	73.1(25.0)	2.856	−3.298	*P* < 0.01
HPA	63.7(28.0)	78.8(23.5)	3.431	−4.396	*P* < 0.001
Independent variable	X(*F* = 4.656, *P* < 0.01)				
Moderator variable	W(*F* = 105.01, *P* < 0.001)				
Interaction	X × W(*F* = 12.810, *P* < 0.001)				
Model 2 (*R*^2^ = 0.154)					
LPA	50.7(27.9)	79.1(18.1)	2.582	−10.978	*P* < 0.001
MPA	63.6(24.5)	73.0(25.0)	2.856	−3.299	*P* < 0.01
HPA	63.8(28.1)	78.9(23.8)	3.431	−4.896	*P* < 0.001
Independent variable	X(*F* = 5.056, *P* < 0.01)				
Moderator variable	W(*F* = 104.408, *P* < 0.001)				
Interaction	X × W(F = 13.569, *P* < 0.001)				

LPA, low physical activity; MPA, moderate physical activity; HPA, high physical activity.

X, PA level; W, Parental co-participation.

Model 1 unadjusted.

Model 2 was adjusted for the characteristics of junior high school students (gender, grade, sibling status, ethnicity) and parental characteristics (level of education).

## 4 Discussion

This study, using a sample of rural junior high school students in China, examines the impact of different levels of PA (low, moderate, high) on HRQoL and explores the moderating role of parental co-participation in this relationship. The results indicate that the higher the level of PA, the higher the students’ HRQoL scores. This finding is consistent with previous studies (29–31), which have demonstrated that PA significantly improves adolescents’ HRQoL (42). In our study, HPA yielded the highest scores across all HRQoL dimensions among rural students, suggesting that HPA has the most pronounced positive effect on the quality of life of rural adolescents. MPA also showed significant positive effects compared to LPA across all HRQoL dimensions, but its impact was somewhat less than that of HPA. While previous literature has studied the effects of PA on adolescents’ HRQoL (8), few have evaluated the variations in these effects based on different levels of PA, especially among rural adolescents. One study by Merglen assessed this, showing that in urban adolescent populations, approximately 14 h of PA per week has been shown to optimize well-being scores, whereas exceeding 17.5 h per week is associated with a reversal of this beneficial effect (43). In contrast to this urban sample, our rural cohort did not demonstrate such a reversal at high PA levels. This discrepancy may be explained by differences in the nature of PA, with rural adolescents engaging more frequently in labor-based rather recreational PA. In another study, participation in three to five different PA was associated with higher well-being (43). More generally, our results confirmed a strong correlation between HRQoL dimensions and PA performance. The more PA adolescents engage in, the more they report improved physical and mental health, social interaction, and academic performance (44). There is strong evidence in the literature regarding the positive impact of higher PA levels on physical functioning, including improving internal and external symptoms and reducing the risk of depression, anxiety, and substance abuse among adolescents (16, 45).

However, the relationship between PA intensity and mental health remains contentious. While some results indicate that higher PA levels bring better mental health, recent studies suggest that low and moderate-intensity activities significantly reduce depression and anxiety, while high PA levels show no effect (46, 47). Another important finding in our data is the link between high PA and school functioning. The literature on this relationship is less clear, although there is well-established evidence of a positive correlation between aerobic fitness and academic performance in school-aged children (48). One possible explanation for this association lies in the impact of aerobic activity on hippocampal volume, the brain region involved in memory processes (49). These findings underscore that PA contributes not only to adolescents’ physical health but also positively affects their psychological and social functioning. During the growth phase, PA fosters social interaction, boosts confidence, and improves school performance, holistically promoting HRQoL. However, the relationship between PA levels and HRQoL may be influenced by various family factors (17, 18), with parental co-participation being one of the key factors. Our study found that parental co-participation moderates the relationship between PA and overall HRQoL. The results show that parental co-participation significantly enhances the positive effects of PA on total HRQoL, especially under HPA, where the effect of parental co-participation is even more pronounced.

According to social capital theory, good social support has positive effects on individual health (50), and the family, particularly parents, is a key source of social support for children and adolescents, playing a critical role in their socio-emotional development (51–53). Parental co-participation, through providing social support, emotional encouragement, and acting as role models, parents help strengthen adolescent’s social capital, thereby amplifying the psychosocial benefits of PA (21–23). One study found that the more frequently parents accompany or participate in PA with their children, the higher the children’s HPA, allowing them to experience the benefits of HPA more fully under parental co-participation (54).

We found that HPA was associated with better HRQoL. Moreover, studies have shown that students who receive parental co-participation in their daily lives tend to have higher levels of HRQoL in psychosocial functioning, physiological functioning, living environment, and overall life satisfaction compared to those who are accompanied by grandparents or others (55). Parental co-participation may have positive effects on children’s physical and mental health, as well as their social adaptation skills (56, 57). In rural areas of China, the PA levels of adolescents are generally lower due to the lack of sports facilities and opportunities for PA. Rural adolescents tend to rely more on outdoor activities and daily labor for PA compared to their urban counterparts (24, 25). Moreover, rural parents’ emphasis on their children’s PA varies due to economic conditions and educational resources (25). The family structure in rural areas is relatively traditional, with parents playing a significant role in their children’s lives. Parental co-participation not only provides emotional support for adolescents but also helps them better cope with the challenges of PA, thereby further enhancing their HRQoL. These findings align with existing research, which highlights that support within the family environment, especially active involvement and companionship from parents, is a critical factor in maximizing the benefits of PA on adolescent health.

This study makes notable contributions to the existing literature in both theoretical and practical aspects. Theoretically, it fills the gap in research regarding the impact of different levels of PA on the HRQoL of rural adolescents. Previous studies have primarily focused on urban adolescents or specific groups, with limited attention given to rural adolescents. By differentiating low, moderate, and high levels of PA, this study systematically investigates the specific effects of each level on HRQoL, thereby enriching the theoretical framework of health behavior. Additionally, this study is the first to empirically validate the moderating role of parental co-participation in the PA-HRQoL relationship, extending social capital theory and emphasizing the critical role of family support in adolescent health development. Practically, this study provides empirical evidence for public health policies, educational interventions, and health promotion programs. In rural areas, parental co-participation in PA not only effectively enhances adolescents’ PA levels but also improves their HRQoL through emotional and social support mechanisms. Policymakers should integrate family participation into health promotion strategies and design family-based intervention programs. Schools and communities can further promote the comprehensive physical and mental health development of adolescents through parent–child activities.

### 4.1 Study limitations

Despite the significant findings of this study, there are several limitations. First, the cross-sectional design of the study limits the ability to infer causality. Future studies should adopt a longitudinal design to further explore the dynamic relationship between PA and HRQoL. Second, the specific content and quality of parental co-participation may influence the moderating effect. For example, whether parents actively participate in PA, provide emotional support, or model behavior may all impact the outcomes. Future research should delve deeper into these specific dimensions to better understand the mechanisms of parental co-participation. This study offers empirical evidence on the association between physical activity and health-related quality of life among rural adolescents, highlighting the moderating role of parental co-participation. These findings lay the groundwork for longitudinal research and the development of parent–child interventions to inform family-centered health promotion strategies in rural settings.

## 5 Conclusion

This study found that PA has a significant positive impact on the HRQoL of rural junior high school students, with higher PA levels leading to greater improvements in HRQoL. Parental co-participation plays a key moderating role in this relationship, particularly under HPA, where the presence of parents enhances the positive effects on HRQoL. These findings highlight the importance of family support in promoting adolescent health. The school can leverage the existing rural community structure to organize parent–child sports events, encouraging parents to participate in physical activities together, this can help increase the frequency of parental involvement and thereby better promote the physical and mental development of adolescents.

## Data Availability

The original contributions presented in the study are included in the article/Supplementary material, further inquiries can be directed to the corresponding authors.
