# Supplementary material for: Effects of different levels of physical activity on the health-related quality of life among rural junior high school students in China: the moderating role of parental co-participation in physical activities
Source: Front Public Health. 2025 May 23;13:1556246. doi: 10.3389/fpubh.2025.1556246 (PMC12141325; doi:10.3389/fpubh.2025.1556246)
Supplement: Supplementary file 2 [file Table_2.docx]

**SUPPLEMENTARY MATERIALS**

**Effects of different levels of physical activity on the health-related quality of life among rural junior high school students in China: The moderating role of parental companionship**

**Weili Yang^1,2*^ , Zhiyun Zhao^2,3^ , Pengcheng Gao^4^ , Xiaodan Guo^1^, Xuguang Jia^5^ and Marcin Białas^1*^**

^1^ Doctor school, Gdansk University of Physical Education and Sport, Gdansk,Poland

^2^ School of Sports and Health,Yibin University, Yibing , China

^3^ School of Education, University of Malaya, Kuala Lumpur, Malaysia

^4^Postgraduate School, Pukyong National University, Pusan, Korea

^5^ The Third People's Hospital of Yibin, Yibing, China

*** Correspondence:**Weilin Yang
Yang.Weilin@awf.gda.pl

Marcin Białas

Marcin.bialas@awf.gda.pl

**Supplementary File**

**File 1**.Internal consistency of the IPAQ (Short Forms) by PA levels

| **PA intensity** | **Cronbach Alpha** |
| --- | --- |
| Low (<600METs/week) | 0.79 |
| Moderate (600-3000METs/week) | 0.87 |
| High (>3000METs/week) | 0.91 |
| Total | 0.85 |

PA: physical activity; IPAQ: international physical activity questionnaire. A value of Cronbach alpha between 0.7 and 0.9 represented satisfactory internal consistency reliability

**File 2**. Internal consistency of Chinese mandarin Children self-reported Version of the PedsQL^TM^

| **Dimensions∕Total** | **Cronbach’s alpha (α)** | **McDonald’s omegas (ω)** |
| --- | --- | --- |
| Emotional functionin | 0.81 | 0.81 |
| Social functioning | 0.69 | 0.69 |
| School functioning | 0.79 | 0.79 |
| Physical health | 0.74 | 0.74 |
| Total score | 0.86 | 0.86 |

A value of Cronbach alpha between 0.69 and 0.86 represented satisfactory internal consistency reliability
